# The intratumoral microbiome: a review of the tumor microenvironment’s fourth axis shaping anti-tumor immunity, cancer prognosis, and therapeutic response

**DOI:** 10.3389/fimmu.2026.1820477

**Published:** 2026-06-04

**Authors:** Joseph G. L. Hunter, Alexandra Roach, Lizbeth Nieves, Ariana Islas, Andrew Islas, Michelle DiPalma

**Affiliations:** 1 School of Life Sciences, Arizona State University, Tempe, AZ, United States; 2 Department of Medicine, University of Arizona College of Medicine, Phoenix, AZ, United States

**Keywords:** cancer, cancer prognosis, immune modulation, immunotherapy, intratumoral microbiome, microbiome, tumor microenvironment (TME), tumor-resident microbes

## Abstract

The tumor microenvironment (TME) is a well-documented, complex, and dynamic ecosystem traditionally considered among three major axes: 1) malignant cells, 2) stromal cells, and 3) infiltrating immune cells; all whose interactions have been assumed to shape tumor growth, progression, and therapeutic response. Specific TME profiles are strongly associated with cancer prognosis, especially those characterized by distinct immune cell populations. Within the TME, less-studied populations of various microorganisms constitute the intratumoral microbiome. While the systemic microbiome is well-established as a regulator of overall health, disease progression, and cancer prognosis, the role of microorganisms residing directly within tumors remains largely underexplored and often overlooked. As evidence on the intratumoral microbiome continues to emerge, it is increasingly apparent that, in addition to the previously characterized components of the TME, these tumor-resident microbes may significantly impact the TME through complex interactions, effectively constituting a fourth component that must be considered alongside malignant, immune, and stromal cells. We therefore propose a framework in which the TME is defined across four axes: malignant cells, stromal cells, infiltrating immune cells, and intratumoral microbes. Herein, we review the effects of intratumoral microbes on various immune cell types commonly studied within the TME, their effects on specific cancers, and subsequent therapeutic insights that arise from understanding the intratumoral microbiome.

## Introduction

The tumor microenvironment (TME) is a complex and dynamic tissue ecosystem that has traditionally been considered to be composed of tumor cells, infiltrating immune cells, and stromal cells of the extracellular matrix (1–3). It is shaped and sustained by a complex series of signaling networks that promote tumor progression by developing resistance to intrinsic anti-cancer responses, as well as to therapeutic anti-cancer responses, such as radiotherapy, chemotherapy, or immunotherapy. These signaling pathways can vary even within the same tumor, further compounding the complexity seen within the TME and complicating predictions of therapeutic response (4). Despite significant heterogeneity, several general trends within solid TME have been elucidated, including gradients that flow from the TME’s outer edge toward its center. For example, oxygen levels are close to physiological norms at the outer edge of the TME, whereas they progressively decrease within the TME, contributing to a hypoxic environment. The pH of the TME similarly follows an outer-to-inner gradient, from physiological pH to acidic pH (5). Of note, these two factors may be responsible not only for immune cell senescence around and in the TME but also play a critical role in small molecule and immunotherapy resistance of certain tumors, as the acidic pH can destabilize binding and even result in reduced stability of antibodies and other proteins used to target tumors (6). In addition to the oxygen and pH gradients, poor nutrient loads, anti-inflammatory cytokines, and the buildup of metabolic by-products can be observed within the TME along similar gradients; however, it is important to note that the TME is highly complex, with pockets of activity where these generalized gradients may not apply (5).

There are several proposed ways to identify subtypes of TME, with two routinely cited in the literature. In 2018, *Thorsson* et al. conducted tumor immunotyping using 10,000 samples of 33 cancer types, which resulted in the following defined TME subtypes: wound healing (C1), IFN-*γ* dominant (C2), inflammatory (C3), lymphocyte-depleted (C4), immunologically quiet (C5), and TGF-β dominant (C6) (7). This TME subtyping has been used by several other groups conducting large-scale immunotyping as well as by groups studying the impact of TME subtypes on tumor therapeutic success (8, 9).

However, in 2021, *Bagaev* et al. conducted TME classification using transcriptomic analysis of >10,000 tumors across 20 cancers, resulting in four TME subtypes that correlate with patient response to immunotherapy. These four TME subtypes were defined as Immune-Enriched/Fibrotic (IE/F), Immune-Enriched/Non-Fibrotic (IE), Fibrotic (F), and Desert (D) (10). The major differentiator from the more established C1-C6 TME subtyping is that the *Bagaev* et al. subtyping focuses primarily on correlating immunotherapy efficacy with the subtypes, potentially at the expense of some of the immunological details provided by the *Thorsson* et al. subtyping.

While subtyping of the TME is important, a universally standardized framework has yet to be established, reflective of the extensive intratumoral heterogeneity that characterizes most tumors. Furthermore, the goal of subtyping differs between academic studies, which focus on understanding the mechanisms underlying the TME, and clinical studies, which focus on identifying markers that may differentiate a tumor as a responder versus a non-responder to treatment. Confounding efforts to subtype the TME is the fact that the intratumoral microbiome, which comprises various microorganisms, including bacteria, fungi, viruses, archaea, and parasites, has been far less studied than the TME’s immune components, until recently. Emerging evidence indicates that the systemic microbiome and its dysbiosis can significantly impact disease outcomes in several ways, including altering immune cell function, influencing disease progression, and affecting patient responsiveness to various treatments (11–15). Similar to dysbiosis of the systemic microbiome, which is associated with worse outcomes for many diseases, dysbiosis of the intratumoral microbiome is also, at times, associated with worse outcomes for patients’ cancer prognosis (15, 16). The lack of integration of the intratumoral microbiome into current TME classification models makes it difficult to use any classification system when discussing intratumoral microbiome/TME interactions. However, for this review, we will focus on the methodology of *Thorsson* et al. when relevant, as it highlights the molecular factors that the intratumoral microbiome may affect.

In this review, we examine how the intratumoral microbiome and microenvironment are entwined, highlighting mechanistic evidence demonstrating how tumor-resident microbes influence major cellular compartments within the TME, including their impact on each major cell type involved in the TME and subsequent effects on specific cancer types. We demonstrate, through a review of intratumoral microbiomes across these cell types, that, in the future, subtyping of the TME would benefit from the inclusion of tumor microbiome analysis, especially if the goal is to differentiate responders from non-responders. We further discuss the intratumoral microbiome and its impact on the progression and risk of specific cancer types. Finally, we extend the insights gained from these studies to explore the potential development of therapeutic strategies that leverage our current understanding of the tumor microbiome.

## Intratumoral microbiome effects on TME immune cells

Intratumoral microbiota are recognized as consistent, low-biomass communities that localize within both malignant cells and immune cells across multiple human tumor types (17). These microbiota and their metabolites shape the tumor immune landscape by skewing effector versus regulatory programs, altering cell polarization, and modulating responsiveness to immunotherapies, including immune checkpoint blockade (ICB), thereby influencing tumor progression and treatment outcomes (18).

In some settings, intratumoral communities promote innate and adaptive immune suppression, whereas specific taxa enhance cytotoxic T-cell infiltration and immunotherapy outcomes (19). Below, we review the primary cells involved in the TME and the impact the intratumoral microbiome, and, in some cases, the systemic microbiome, has on these cell types (18).

### CD8^+^ T cells

Cytotoxic CD8^+^ T cells are central mediators of antitumor immunity, and accumulating evidence demonstrates that the intratumoral and systemic microbiome can either erode or enhance their function, depending on the composition and metabolic activity of local microbial communities (18).

**Figure 1:**
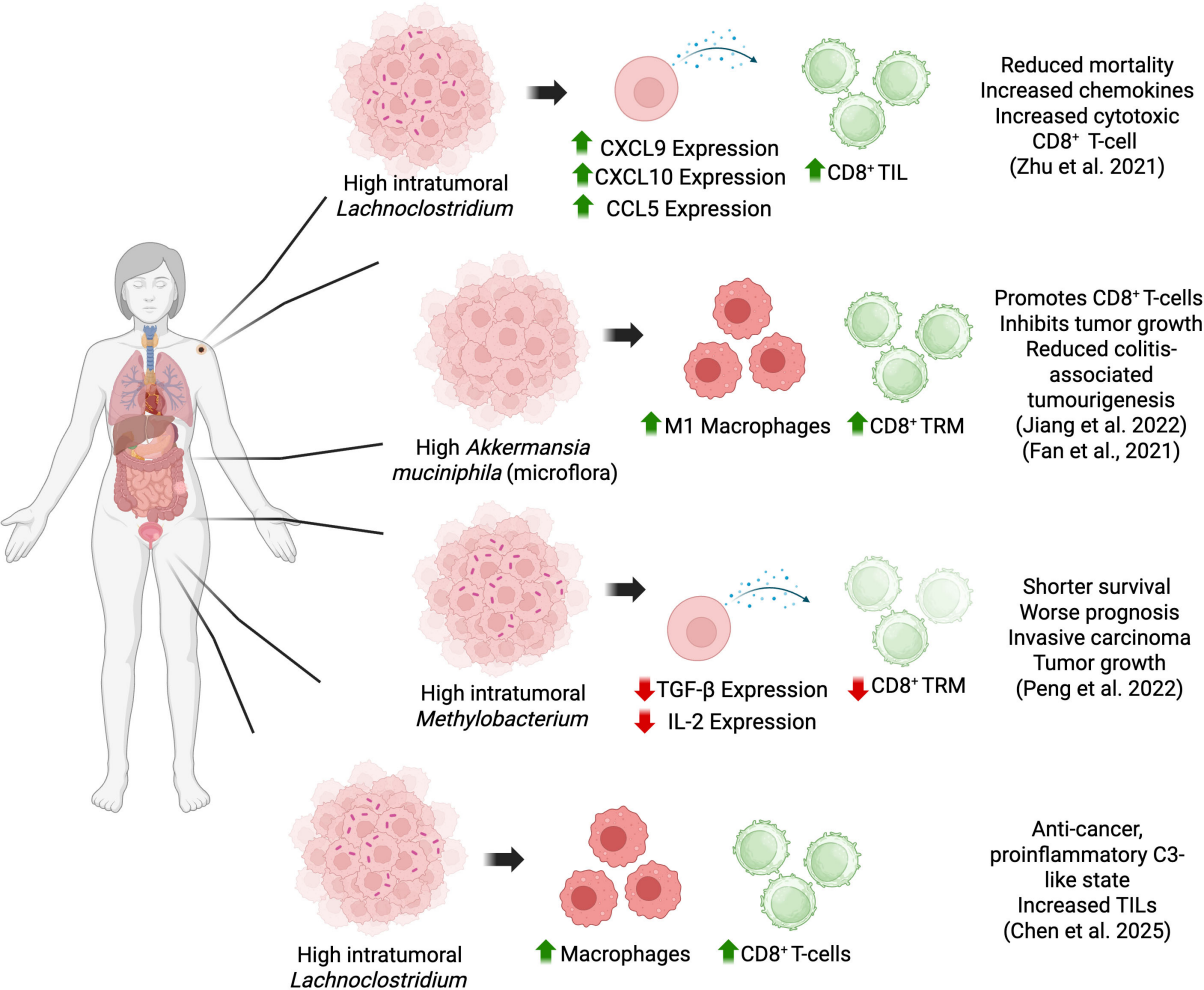
Schematic representation of how distinct intratumoral bacteria can modulate the tumor immune microenvironment. Higher presence of *Lachnoclostridium* species in subcutaneous melanoma has been associated with increased chemokine expression (CXCL9, CXCL10, CCL5), enhanced tumor-infiltrating CD8^+^ T-cells, and reduced mortality. Similarly, high microfloral *Akkermansia muciniphila* in patients with intestinal cancer was associated with higher M1 macrophage polarization and the recruitment of CD8^+^ tissue-resident memory (TRM) T-cells, contributing to improved tumor control and reduced colitis-associated tumorigenesis. In contrast, high intratumoral *Methylobacterium* correlates with decreased TGF-β and IL-2 expression, reduced CD8^+^ TRM T-cells, and tumor progression, resulting in worse patient prognosis.

In gastric cancer, colonization of tumors by *Methylobacterium* has been associated with downregulation of TGF-β, affecting CD8^+^ tissue-resident memory cell frequency, suggesting a potential role in local immune modulation (20) (**Figure 1**). In contrast, beneficial microbes such as *Akkermansia muciniphila*, which are present in the intestinal microbiota, have been associated with remodeling of the TME toward a pro-inflammatory, C3-like state (21).

In colorectal cancer *A. muciniphila* in the intestinal microflora was shown to be associated with activation of TLR2/NF-κB/NLRP3 signaling and the promotion of accumulation of M1-like tumor-associated macrophages in the TME, both *in vitro* and *in vivo* (22, 23). This species has also been shown to enhance CD8^+^ T-cell recruitment into the microenvironment and effector function, reducing tumor volume *in vivo* as well as downregulating PD-L1, increasing IL-2 and IFN-γ levels, and increasing CD8^+^ T-cell proliferation *in vitro* (23).

In cutaneous melanoma, intratumoral bacterial peptides derived from 41 distinct species were found to be presented on HLA-I and HLA-II molecules by both tumor cells and antigen-presenting cells, thereby expanding the repertoire of immunogenic epitopes accessible to CD8^+^ T cells and enhancing tumor-infiltrating lymphocyte IFN-γ secretion, driving a C2-like TME (20). Large-scale analyzes in melanoma further reveal that sixteen intratumoral bacterial genera, particularly *Lachnoclostridium*, correlated positively with tumor infiltration by CD8^+^ T cells alongside increased expression of chemokines CXCL9, CXCL10, and CCL5, indicating active recruitment of cytotoxic immune cells (21).

*Lachnoclostridium* was further studied in bladder urothelial carcinoma. It was found to increase the release of chemokines that drive the recruitment of macrophages and CD8^+^ T cells, thereby driving the TME toward an anti-cancer, proinflammatory C3-like state (24). In cervical cancer, tumors with higher *Bifidobacteriaceae* levels showed increased recruitment and activation of CD8^+^ cells, which correlated with improved outcomes in patients who received radiotherapy, demonstrating that the intratumoral microbiome is not only significant for immunotherapy success but also for other therapeutic approaches (25).

Experimental evidence demonstrates that microbial metabolites further modulate CD8^+^ T-cell activity. In mouse melanoma models, translocation or intratumoral administration of *Lactobacillus reuteri* increased the type-1 cytotoxic phenotype via indole-3-aldehyde-dependent activation of the aryl hydrocarbon receptor (AhR), significantly boosting IFN-γ secretion, promoting a type-1 cytotoxic, and impairing tumor growth (26). Several intratumoral communities also display synergistic interactions with immunotherapy. *Bifidobacterium* species in MC38 colorectal cancer tumors and *Akkermansia* in melanoma were shown to promote STING-dependent cytokine release in mouse models, with the potential to promote NK-DC crosstalk and macrophage reprogramming toward antitumor phenotypes that reinforce CD8^+^ cytotoxicity (19, 27) (**Figure 1**).

### Natural killer cells

In preclinical models, intratumoral microbes have been shown to modulate NK cell activity and other immunological functions, thereby affecting tumor growth and progression, depending on microbial composition and tumor type (28). *Fusobacterium nucleatum* remains a prototypic example of a microbe that promotes immune evasion. *In vitro* and co-culture systems have shown that Fap2 lectin binds the inhibitory receptor TIGIT (T cell immunoreceptor with Ig and ITIM domains) on NK cells and T lymphocytes, suppressing their cytotoxic activity (29). Furthermore, interactions involving CEACAM1 have been implicated in suppressing NK-cell cytotoxicity and T-cell responses, potentially contributing to tumor shielding from immune attack when *F. nucleatum* is present in the intratumoral microbiome (29).

The NK cell-microbiome axis extends beyond bacteria to include intratumoral viruses, which can strongly influence NK infiltration and patient prognosis. In human soft tissue sarcomas, the intratumoral viral microbiome showed a robust positive correlation with NK cell infiltration (29). Tumors enriched with viral sequences exhibited significantly higher NK cell density and improved metastasis-free overall survival (29, 30). These data indicate that, beyond consideration of bacterial populations within a tumor, other microbial components, such as viruses, can act as potent NK-activating signals within the intratumoral ecosystem, promoting immunosurveillance and shaping clinical outcomes.

Similarly, analyzes in esophageal squamous cell carcinoma (ESCC) reveal an inverse relationship between intratumoral bacterial diversity and NK infiltration. Higher Shannon diversity (a measure of microbial diversity within a community) and enrichment of *Lactobacillus* correlated with reduced NK-cell presence, increased PD-L1^+^ epithelial cells, and expanded tumor-associated macrophages, collectively forming an immunosuppressive microenvironment that constrains NK-cell cytotoxic function (31).

Innate immune signaling pathways serve as key intermediaries, linking intratumoral microbes to NK-cell activation. STING and IFN-I signaling, which can be stimulated by microbial DNA, have been shown to drive the expression of IL-15, IL-18, and CXCL10 in the TME, potentially facilitating NK recruitment, survival, and activation (18, 32). Intratumoral *Akkermansia muciniphila* and *Bifidobacterium* species amplify these pathways, increasing NK density and enhancing crosstalk with dendritic cells and macrophages to promote antitumor cytotoxicity, particularly in melanoma and colorectal cancer mouse models (31).

In lung and gastrointestinal cancers, chronic microbial stimulation of IL-1β and IL-23 production by myeloid cells fostered IL-17–driven inflammatory circuits dominated by neutrophils and γδ T cells, creating suppressive niches that may restrict NK function and shift the balance of innate immunity toward tumor progression (18).

### CD4^+^ T cells

CD4^+^ T-cells differentiate into many subsets, including Th1, Th2, Th17, and T follicular helper (Tfh) subsets, as well as Tregs, which are discussed in detail in the following subsection (33). The Th1 subset promotes anti-tumor immunity by secreting pro-inflammatory cytokines, such as IFN-γ, thereby activating CD8^+^ T cells and M1 polarized macrophages, both of which mediate anti-tumor effector functions (34). The Th2 subset promotes humoral immunity and tissue repair and is frequently linked to immunosuppressive and pro-tumorigenic activities within the TME (35). The Th17 subset is capable of driving neutrophil-mediated inflammation, while the Tfh subset supports the generation of high-affinity antibodies via B-cell help (33). Evidence across multiple tumor types reveals how the intratumoral microbiome differentially modulates CD4^+^ T-cell lineages and their effector functions.

In ovarian cancer, intratumoral microbes stratify tumors into immune-enriched and immune-deficient phenotypes, with the immune-enriched subtype showing higher CD4^+^ T-cell activity, elevated Th1 expression, and improved survival (36). Distinct intratumoral bacterial species correlate with cytokines associated with Th1 and M1 macrophage polarization, underscoring functional coordination between CD4^+^ helper states and the TME’s microbial composition (37).

The intratumoral microbial ecosystem has the potential to shape other CD4^+^ T-cell subsets as well. Microbial antigen mimicry in glioblastoma was found to enable bacterial peptides to be presented on HLA-II molecules, thereby enhancing CD4^+^ T-cell activation, cytokine production, and CD4^+^ T-cell-mediated antitumor responses (38).

In colorectal cancer models colonized with *Helicobacter hepaticus*, intratumoral bacteria were found to drive Tfh CD4^+^ T-cell expansion and the maturation of tertiary lymphoid structures, thereby enhancing antigen presentation, facilitating lymphatic trafficking, and generating a microenvironment favorable to CD4-dependent antitumor immunity (39). Several tumor-resident microorganisms have been found to suppress Th1 immunity or promote Th2/Treg-biased environments. Viral constituents of the intratumoral microbiome, including EBV and HBV, were found to downregulate IFN-γ and remodel helper T-cell networks, thereby inhibiting antitumor Th1 activation (39).

In diffuse large B-cell lymphoma, microbial signatures are strongly associated with transcriptional programs governing Th1 and Th17 immunity, including cytokine-receptor interactions, T-cell differentiation pathways, and IL-17 signaling, demonstrating that bacterial enrichment can actively rewire helper T-cell polarization within lymphoid malignancies (40).

### Regulatory T cells

Regulatory T cells (Tregs) are key cellular mediators of immunosuppression within the TME as they’re the primary down-regulators of immune responses (33). In esophageal and oral squamous cell carcinoma, *F. nucleatum* colonization was found to promote Treg enrichment in tumor tissues, weakening antitumor immunity and facilitating both persistent bacterial colonization and tumor progression (41, 42). Other intratumoral bacterial communities appear capable of restraining Treg expansion or activity. Certain tumor-resident microbes have been found to enhance PD-1 signaling and intracellular infection response pathways while downregulating FOXO-mediated transcription and IFN-γ programs, a pattern associated with decreased Treg activation and improved effector T-cell function (43).

In ovarian cancer, tumors classified as immunodeficient, characterized by lower T-cell infiltration and worse prognosis, exhibited distinct intratumoral microbial signatures that correlate with reduced M1 macrophage activity and impaired Th1 signaling, conditions permissive for Treg dominance and an immunosuppressive tumor microenvironment (36).

Direct mechanistic evidence also supports microbial induction of Treg differentiation in several cancer contexts. Certain intratumoral microbes, including *Nevskia ramosa* and *Staphylococcus aureus*, were found to promote Treg activation in prostate and liver cancers, thereby facilitating immune escape and disease progression (32, 44).

Fungal constituents of the tumor microbiome can produce similar effects. *Aspergillus sydowii* was shown to enhance lung tumor growth by inducing IL-1β–dependent activation of myeloid-derived suppressor cells, which, in turn, promote expansion of PD-1^+^ exhausted CD8^+^ T cells and Tregs, further suppressing antitumor immunity (45).

Several bacterial taxa have been shown to inhibit Treg activity, thereby promoting effective antitumor responses, opening avenues for potential therapeutic approaches. For example, engineered intratumoral microbial systems reduced Treg abundance while enhancing CD45^+^ leukocyte infiltration, MHC-II expression on dendritic cells, and IFN-γ-producing CD8^+^ T cells, collectively shifting the tumor immune environment toward effector responses (46–48). Depending on microbial community composition, Tregs may either expand to promote tumor immune evasion or be functionally suppressed to permit robust antitumor responses. This dynamic interplay highlights the therapeutic potential of manipulating tumor-resident microbial ecosystems to rebalance tolerance and immunity within the TME.

### B cells

Although less well characterized in the TME than T cells, tumor-associated B cells contribute to antigen presentation, antibody production, and the formation of tertiary lymphoid structures (TLS), thereby serving as important modulators of anti-tumor immunity (49). In diffuse large B-cell lymphoma (DLBCL), tumor-resident microbial communities are tightly linked to host transcriptional programs governing B-cell receptor signaling, cytokine-cytokine receptor interactions, and humoral immune pathways (40).

Of all the cell types reviewed, B cells had by far the least intratumoral microbiome data available, with most studies focusing on the systemic microbiome’s impact on B cells. We believe there is a current gap in research that specifically identifies the mechanisms by which intratumoral microbiomes affect B cells. Many viral diseases that directly affect B cells, such as EBV, suggest that the intratumoral virome may also have a significant impact on the TME in addition to the intratumoral microbiome.

### Macrophages

Macrophages are among the most directly influenced immune-cell populations within the tumor microbiome niche, as intratumoral bacteria frequently localize within CD68^+^ tumor-associated macrophages (TAMs), and microbial components can persist intracellularly long after phagocytosis (17, 50). Several tumor-resident taxa drive macrophage polarization toward pro-tumorigenic M2-like phenotypes. In colorectal cancer, *Fusobacterium nucleatum* was shown to influence M2 polarization and tumor progression via TLR4- and NF-κB pathways. In contrast, its LPS and outer membrane vesicles have been shown to enhance TAM-driven immunosuppression (51, 52).

Pancreatic cancer provides an example of macrophage reprogramming, in which *Proteobacteria*-dominated intratumoral communities were shown to induce tolerogenic myeloid differentiation that suppressed effector T-cell responses (53). By contrast, in colorectal tumors*, Akkermansia muciniphila* was shown to activate TLR2/NF-κB/NLRP3 signaling to promote M1-like macrophage accumulation, supporting an inflammatory, antitumor TME (18).

In ovarian cancer, distinct intratumoral microbial signatures stratified tumors into immune-enriched and immune-deficient subtypes, with the immune-enriched group featuring significantly higher M1 macrophage infiltration (36).

### Dendritic cells

Dendritic cells (DCs) orchestrate T-cell priming and shape both local and systemic antitumor immunity (33). Microbial signals tightly regulate their function within the intratumoral microenvironment in ways similar to macrophage regulation. In colorectal cancer, *Fusobacterium nucleatum* outer membrane vesicles and LPS were shown to activate pro-inflammatory pathways in DCs, yet paradoxically promote tumor progression by sustaining chronic, non-resolving inflammation and by driving the differentiation of immunosuppressive M2 macrophages (54–56).

In melanoma, the relationship between intratumoral microbial antigens and dendritic cell (DC) cross-presentation is becoming increasingly well defined. Bacterial peptides were displayed on both HLA-I and HLA-II molecules by tumor cells and DCs, potentially promoting enhanced activation of CD8^+^ and CD4^+^ T cells and increasing IFN-γ secretion by tumor-infiltrating lymphocytes, driving a C2-like TME (16, 20).

### Neutrophils and other granulocytes

Neutrophils and related granulocytes are highly responsive to microbiome-derived cues and can be functionally reprogrammed to support tumor growth (57). Intratumoral bacteria in oral squamous cell carcinoma and colorectal cancer were shown to activate MAPK and NF-κB pathways, promoting the expansion of myeloid-derived suppressor cells and neutrophils. In this neutrophil-rich, immunosuppressive microenvironment, CTLA-4 and PD-1 were upregulated, and regions of high bacterial load formed physical and functional barriers to T-cell infiltration (58).

In vulvar squamous cell carcinoma, specific tumor-resident taxa such as *Fusobacterium nucleatum* and *Pseudomonas aeruginosa* were shown to be enriched in tumors with higher CD66b^+^ neutrophil infiltration, elevated neutrophil serine protease expression, increased intratumoral microabscesses, and shorter time to tumor progression, directly linking neutrophilic inflammation, bacterial composition, and poor clinical outcome (59).

Large-scale bioinformatic analyzes in gastric cancer demonstrated that intratumoral microbiome signatures are tightly coupled to immune infiltration patterns and prognostic immune subtypes, with specific bacteria associating with distinct immune gene programs and differential responses to chemotherapy and immunotherapy, implying that neutrophil- and other myeloid-modulating microbial communities can shape disease course at a systems level (60).

Conceptual frameworks from recent reviews emphasize that intratumoral microbiota influence multiple metastatic hallmarks, including chronic inflammation, epithelial-mesenchymal transition, and immune modulation, across tumor types, positioning neutrophils as central effectors through which these bacteria orchestrate both pro- and antitumor activities (61, 62).

### Fibroblasts and stromal cells

Fibroblasts and other stromal cells are key organizers of the tumor microenvironment, generating extracellular matrix (ECM), cytokines, chemokines, and mechanical cues that regulate angiogenesis, immune cell trafficking, tissue stiffness, and metastatic niche formation (63). Cancer-associated fibroblasts (CAFs) shape desmoplasia and immune exclusion through factors such as TGF-β, IL-6, CXCL12, MMPs, and dense collagen networks, directly affecting drug delivery and response to immunotherapy (63).

There is also evidence that intratumoral microbes can reprogram fibroblast phenotypes. In colorectal cancer, *Actinomyces* was shown to invade tumor-associated fibroblasts and influence pro-tumor myofibroblastic features through TLR2/NF-κB signaling (64). Gut-derived *Bifidobacterium adolescentis* was shown to generate an anti-tumor CAF subset (CD143^+^) via Wnt/β-catenin–dependent GAS1 induction, suppressing CRC growth (47). Direct bacterial infection can also alter fibroblast signaling. In gastric cancer, *Helicobacter pylori* was shown to induce VCAM-1 in fibroblasts through JAK/STAT1, enabling tumor invasion via αvβ5/1 integrins (65). Oral-associated pathogens, *Fusobacterium nucleatum* and *Porphyromonas gingivalis* are known to disrupt adhesion by driving EMT-like changes through E-cadherin/β-catenin hijacking or MMP-9 induction (66). As major producers of the extracellular matrix, cancer-associated fibroblasts (CAFs) mediate microbe-driven adhesion and matrix changes, which reinforce shifts in tissue stiffness and migratory pathways that may highly affect the TME (64).

Microbial activation of ROS, NF-κB, β-catenin, and TLR pathways has been shown to affect both immune and stromal populations, with fibroblasts contributing to ROS generation that drives DNA damage, EMT, and matrix degradation (67). These inflammatory programs alter fibroblast activation, collagen deposition, and immunomodulatory cytokine production (TGF-β, CXCL12) (68). Microbes can also reprogram fibroblasts indirectly via exosomes from infected tumor cells, which promote angiogenesis, CAF activation, ECM remodeling, and pre-metastatic niche formation (69).

**Figure 2:**
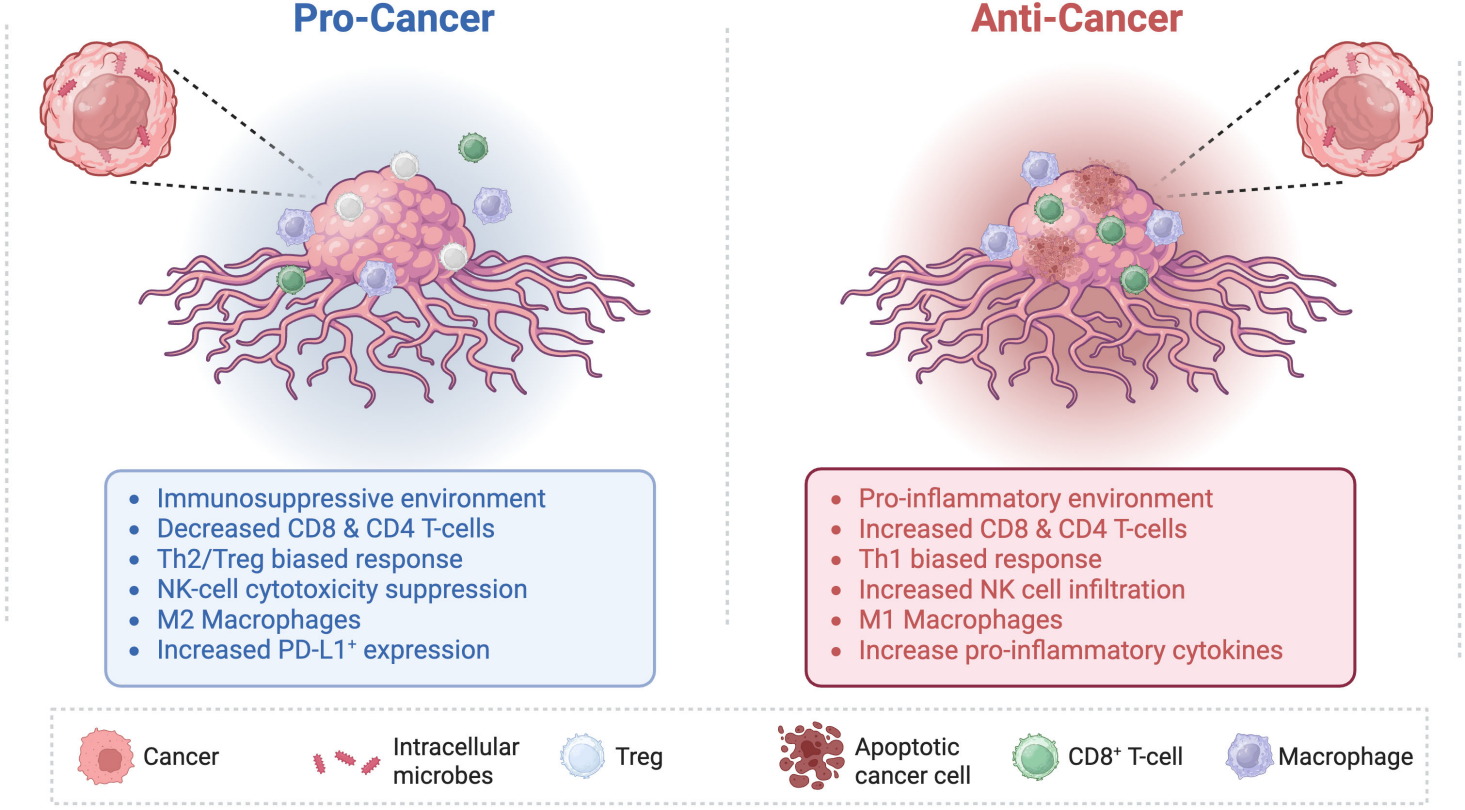
A general overview illustrating the contrasting microbial-induced immune-modulating effects associated with tumor progression (pro-cancer) and tumor control (anti-cancer).

Taken together, the effects described above, largely driven by the composition of the intratumoral microbiome, can significantly influence whether tumor tissue adopts a pro-cancer or anti-cancer state (**Figure 2**). Consequently, the tumor’s overall behavior and progression may be altered.

## The role of the tumor microbiome for specific cancers

Given that the intratumoral microbiome has been shown to significantly influence immune cell function, it is also likely to impact the surrounding tissue microenvironment and associated tumor biology. Using genomic bank and molecular sequencing data, several groups have analyzed the diversity and distribution of bacteria within various tumors to examine how they interact with surrounding tumor cells and involved tissues (17). Furthermore, accumulating evidence suggests that the impact of specific microbial species on tumor biology can be tumor-type specific. Here, we build on our previous explanations of the intratumoral microbiome’s impact on specific immune cells and further provide an overview of the major findings of its impact on specific tumor types (**Table 1**).

**Table 1:** Bacteria reported to be affiliated with tumor development by cancer type.

Cancer type	Associated bacteria (Phylum, Genus, and Species)	Citations
Colorectal Adenocarcinoma	*• Bacteroides fragilis * *• Bifidobacterium breve* *• Candida albicans* *• Campylobacter jejuni* *• Escherichia coli * *• Fusobacterium nucleatum* *• Parvimonas micra* *• Porphyomonas gingivalis* *• Prevotella intermedia* • Alistipes* • Bacillus* • Bilophila* • Blautia* • Bifidobacterium* • Campylobacter* • Clostridiales* • Clostridium* • Dialister* • Erysipelotrichaceae* • Faecalibacterium* • Faecalitea* • Firmicutes* • Mycoplasma* • Mogibacterium* • Parvimonas* • Peptostreptococcus* • Pseudomonadata* • Selenomonas* • Thermus* • Actinobacteria** *• Candida tropicalis†*	(16, 70–79)
Gastric Adenocarcinoma	*• Actinomyces oris* *• Cutibacterium acnes* *• Fusobacterium nucleatum* *• Helicobacter pylori* *• Kytococcus sedentarius* *• Pseudomonas yamanorum* *• Ralstonia insidiosa* *• Streptococcus anginosus* *• Staphylococcus saccharolyticus* • Bacteroides* • Campylobacter* • Dialister* • Lactobacillus* • Massilia* • Methylobacterium* *• Oribacterium* • Photobacterium* • Prevotella* • Veilonella* • Selenomonas* • Streptococcus* • Stomatis*	(16, 69, 70, 73, 80–85)
Intrahepatic Cholangiocarcinoma	*• Paraburkholderia fungorum* • Actinobacteria* • Aerococcus* • Alphaproteobacteria* • Gammaproteobacteria* • Lawsonella* • Massilia* • Paenibacillus* • Porphyrobacter* • Sphingomonas* • Staphylococcus*	(18, 86, 87)
Pancreatic adenocarcinoma (PDAC)	*• Acidovorax ebreus* *• Bacillus clausii* *• Citrobacter freundii* *• Clostridium butyricum* *• Cutibacterium acnes* *• Fusobacterium nucleatum* *• Klebsiella pneumoniae* *• Lactobacillus reuteri* *• Lactobacillus marinus* *• Mycoplasma hyopneumoniae* *• Porphyromonas gingivalis* *• Ralstonia insidiosa* • Actinobacteria* • Bacteroides* • Bifidobacterium* • Deferribacteres* • Fusobacteria* • Gammaproteobacteria* • Lactobacillus* • Mycoplasma* *• Paenibacillus* • Peptoniphilus* • Proteobacteria* • Pseudoxanthomonas* • Saccharopolyspora* • Streptomyces* • Verrucomicrobia* *• Malessezia†*	(16, 70–73, 83, 88–91)
Hepatocellular Carcinoma	*• Akkermansia muciniphila* *• Clostridium scindens* • Actinobacteria* • Bifidobacterium* • Cutibacterium* • Enterobacteriaceae* • Firmicutes* • Fusobacterium* • Neisseria* • Methylobacterium* • Prevotella* • Proteobacteria* • Ruminococcaceae* *• Malessezia†* *• Saccharomyces cerevisiae†*	(11, 71, 73, 92–95)
Lung (Adenocarcinoma and Squamous Cell Carcinoma)	*• Escherichia coli* *• Lactobacillus rhamnosus* *• Pseudomonas putida* *• Ralstonia insidiosa* *• Rothia dentocariosa* *• Staphylococcus aureus* *• Thermostaphylospora chromogena* • Acidovorax* • Actinobacter* • Ancylobacter* • Bacteroides* • Chloroflexus* • Cloacibacterium* • Gemmatimonadetes* • Herbaspirillum* • Mycobacterium* • Parvibaculum* • Prevotella* • Proteus* • Renibacterium* • Sphingomonadaceae* • Streptococcus* • Subdoligranulum* • Veilonella* *• Malassezia restricta†*	(16, 72, 73, 96–98)
Breast Cancer	*• Escherichia coli* *• Enterotoxigenic Bacteroides fragilis* *• Fusobacterium nucleatum* *• Staphylococcus aureus* *• Staphylococcus epidermidis* • Acidibacillus* • Actinobacter* • Actinomyces* • Bartonella* • Citrobacter* • Conexibacteraceae* • Corynebacterium* • Coxiella* • Cytophagaceae* • Enterobacter* • Flavobacteriaceae* • Fusobacterium* • Gluconacetobacter* • Hydrogenophaga* • Lachnoclostridium* • Lactobacillus* • Methylibium* • Neisseria* • Paracoccus* • Prevotella* • Propionicimonas* • Pseudogulbenkiania* • Pseudomonas* • Rickettsia* • Sphingomonas* *• Malassezia restricta†* *• Aspergillus chevalieri†*	(16, 17, 41, 70, 72, 99–104)
Prostate Adenocarcinoma	*• Bradyrhizobium elkanii* *• Bradyrhizobium japonicum* *• Campylobacter concisus* *• Escherichia Coli * *• Herminiimonas arsenicoxydans* *• Lactobacillus crispatus* *• Listeria monocytogenes* *• Methybacterium radiotolerans* *• Ochrobactrum anthropi* *• Pedicoccus pentosaceus* *• Pseudoarthrobacter chlorophenolicus* *• Pseudomonas aeruginosa* *• Stenotrophomonas maltophilia* *• Streptococcus pneumoniae* *• Thermos thermophilus* *• Xanthomonas albilineans* • Enhydrobacter* • Lautropia*	(105, 106)
Cervical Squamous Cell Carcinoma	*• Atopobium vaginae* *• Chlamydia trachomatis* *• Escherichia coli* *• Fusobacterium necrophorum* *• Fusobacterium nucleatum* *• Lactobacillus iners * *• Trichomonas vaginalis* *• Ureaplasma parvum* *• Ureaplasma urealyticum* • Alistepes* • Atopobium* • Bacillus* • Bidifobacteria* • Lactobacillus* • Mycobacterium* • Neisseria* *• Prevotella* • Pseudomonas* • Sneathia* • Staphylococcus* *• Malassezia restricta†*	(25, 39, 70, 71, 73, 107, 108)
Esophageal Cancer	*• Escherichia coli* *• Fusobacterium nucleatum* *• Prevotella melaninogenica* *• Prevotella oris* *• Porphyromonas gingivalis* *• Streptococcus anginosus* *• Veillonella parvula* • Bacteriodes* • Blautia* • Campylobacter* • Curvibacter* • Enterobacteria* • Faecalibacterium* • Lactobacillus* • Nitrobacter* • Planckia* • Siphonobacter* • Streptococcus*	(31, 70, 73, 109–112)
Head and Neck Squamous Cell Carcinoma	*• Capnocytophaga canimorsus* *• Clostridium perfringens* *• Fusobacterium nucleatum* *• Porphyromonadaceae bacterium* *• Porphyromonas gingivalis* *• Prevotella intermedia* *• Simonsiella muelleri* *• Streptococcus cristatus* *• Streptococcus gordonii* *• Streptococcus mutans* *• Streptococcus sp. HMSC056D07* *• Tissierellia bacterium* *• Veillonella parvula* *• Alloprevotella* • Fusobacteria* • Prevotella* • Streptococcus*	(70, 73, 109, 113–120)
Renal Cell Carcinoma	*• Cutibacterium acnes* *• Staphylococcus epidermitis* • Alicyclobacillus* • Babesia* • Colletotrichum* • Cyanobacteria* • Cytobacillus* • Deinococcus* • Gluconobacter* • Leuconostoc* • Parabacteriodes* • Plasmodium* • Toxoplasma* • Verrucomicrobium* *• Saccharomyces cerevisiae†* *• Malassezia restricta†*	(73, 121–123)
Adrenocortical Carcinoma	*• Prevotella oris* *• Veillonella parvula* • Proteobacteria* • Pseudomonas* • Serratia*	(73, 124)
Ovarian cysadenocarcinoma	*• Acinetobacter seifertii* *• Achromobacter deleyi* *• Bifidobacterium subtile* *• Buchnera aphidicola* *• Chlamydia trachomatis* *• Comamonas aquatica* *• Corynebacterium jeikeium* *• Fusobacterium nucleatum* *• Gemmatirosa kalamazoonesis* *• Klebsiella michiganensis * *• Microcella alkaliphila* *• Paraburkholderia edwinii* *• Propionibacterium acnes* *• Staphylococcus epidermitis* *• Veillonela nakazawae* • Brachymonas* • Erwinia* • Firmicutes* • Halolamina* • Lactobacillus* • Lactococcus* • Luteimonas* • Magnetospirillum* • Mitsuokella* • Proteobacteria* • Saliniesphaera* • Simiduia* • Streptococcus* • Terasakiella* *• Malassezia†*	(16, 36, 72, 125)
Uterine and Endometrial carcinoma	*• Atopobium vaginae* *• Escherichia coli * • Accumulibacter* • Bacteroides* • Blautia* • Caldimonas* • Campilobacterota* • Candidatus* • Derxia* • Enterococcus* • Firmicutes* • Fusobacterium* • Gardnerella* • Nitriliruptor * • Nonlabens* • Pelomonas* • Porphyromonas* • Prevotella* • Roseiflexus* • Shigella* • Steptosporangium* • Zavarzinella* • Zooshikella*	(73, 74, 100, 101)
Urothelial carcinoma	*• Acinetobacter baumannii* *• Escherichia coli * • Acidbacillus* • Acinetobacter* • Bacillus* • Cyanothece* • Lachnoclostridium* • Lactobacillus* • Mycobacterium* • Terrabacter* *• Malassezia restricta†*	(24, 73, 101, 126)
Cutaneous Melanoma	*• Acinetobacter cumulans* *• Actinomyces odontolyticus* *• Anaerostipes kyriazirhamnosivorans* *• Bacteroides ovatus* *• Bacteroides vulgatus* *• Brachybacterium alimentarium* *• Campylobacter concisus* *• Campylobacter lanienae* *• Campylobacter showae* *• Clostridium celerecrescens* *• Clostridium clostridioforme* *• Clostridium ramosum* *• Corynebacterium afermentans* *• Corynebacterium humireducens* *• Corynebacterium kefirresidentii* *• Cutibacterium acnes* *• Cutibacterium granulosum* *• Dialister invisus* *• Dialister pneumosintes* *• Eikenella corrodens* *• Enterococcus faecalis* *• Enterococcus faecium* *• Enterobacter cancerogenus* *• Enterobacter hormaechei* *• Escherichia coli* *• Fusobacterium nucleatum* *• Gardnerella vaginalis* *• Gemella haemolysans* *• Klebsiella oxytoca* *• Kingella denitrificans* *• Kineothrix alysoides* *• Lactobacillus johnsonii* *• Lactobacillus reuteri* *• Leptotrichia hongkongensis* *• Leuconostoc inhae* *• Paracoccus marcusii* *• Photobacterium rosenbergii* *• Porphyromonas bennonis* *• Prevotella buccalis* *• Schaalia odontolytica* *• Selenomonas artemidis* *• Shewanella decolorationis* *• Sphingomonas dokdonensis* *• Sphingomonas kyeonggiensis* *• Sphingomonas melonis* *• Sphingomonas roseiflava* *• Staphylococcus aureus* *• Staphylococcus capitis* *• Staphylococcus caprae* *• Staphylococcus lugdunensis* *• Streptococcus gordonii* *• Streptococcus salivarius* *• Veillonella dispar* *• Veillonella parvula* • Algibacter* • Collinsella* • Epilithonimonas* • Firmicutes* • Fusobacterium* • Lachnoclostridium* • Proteobacteria* • Porphyromonas* • Trueperella* *• Fusarium fujikuroi†* *• Malassezia restricta†*	(16, 20, 72, 115, 127, 128)
Glioblastoma Multiformae	*• Fusobacterium nucleatum* • Actinobacteria* • Burkholderiales* • Firmicutes* • Lactobacillus* • Proteobacteria* • Rhizobiales* *• Fusarium fujikuroi†* *• Malassezia restricta†*	(16, 38, 73, 129)

(*) indicates Phylum or Genus level classification, (†) indicates a fungi.

### Gastrointestinal and colorectal cancer

The impact of the microbiome on Gastrointestinal (GI) and Colorectal Cancer (CRC) has been extensively studied compared to other tumor types, in large part due to the high concentration of bacteria, both commensal and pathogenic, present in the digestive tract. Traditional estimates assume at least 400–500 unique bacterial species live in this space, with modern genetic sequencing increasing that estimate to up to 3,000 species (30). In patients with colorectal cancer, evidence has been found that dysbiosis of the colon microbiome is associated with an impact on colon cancer risk, which is correlated with reduced diversity within CRC tumors in comparison to standard colon tissue (30, 130). Within the tumor microbiome, the presence of *Fusobacterium nucleatum, Bactoides fragilis*, and *Escherichia coli* has been shown to influence tumor growth directly (30, 70). *Fusobacterium nucleatum* concentration is associated with decreased recruitment of anti-cancer tumor-infiltrating T-cells, allowing for increased tumor growth and increased production of proinflammatory cytokines, which appear to correlate with increased metastasis of colorectal cancer. *Bacteroides* is associated with the recruitment of other pathogenic bacteria into the tumor microbiome, resulting in increased inflammation within the tumor (71) while *Escherichia* coli is associated with increased DNA alkylation, increasing the rate of potential mutation in cancer cells (131).

Additionally, *Bacteroides fragilis* and *Erysipelotrichaceae* have both been associated with immunological cell death within the tumor, suggesting a potential associated reduction in the impact of targeted chemotherapy. In one taxonomic composition analysis comparing tumor tissue and matched non-cancerous colon tissue, tumor tissue had a higher concentration of *Fusobacteriota* and *Pseudomonadata.* In comparison, the colon tissue had a higher concentration of *Verrucomicrobiota.* Further, both tissues showed similar concentrations of *Actinobacteriota*, *Bacillota, Mycoplasmatota*, and *Bacteroidetes*, suggesting a relationship between the proportions of these bacterial groups in the tissue and the potential for tumorigenesis (70–72).

### Gastric cancer

At the tissue level, *Helicobacter Pylori* has been well established to increase the risk of gastric adenocarcinoma, indirectly through increased local inflammation, and directly, by the release of virulence factors from this pathogen into at-risk tissue to increase signaling in multiple cellular pathways that increase cell proliferation, including PI3-kinase/Akt, Jak/Stat, and Ras/Raf/ERK, among other pathways, resulting in tumorigenesis (132–134). Within the intratumoral microbiome, *H. pylori* has been shown to remain a significant contributor to the development of gastric adenocarcinoma. At the same time, the intratumoral microbiome also showed increased abundance of *Enterococcus faecium* and *Micrococcus luteus* (80). Further, an increased intratumoral concentration of *Staphylococcus saccharolyticus* was associated with increased methylation of the ZNF215 gene in cancer cells, which correlated with increased proliferation and metastasis of these cells (80). Separately, changes in the bacterial concentration within the overall tissue microbiome have also been linked with the development of adenocarcinoma. In one study, the concentrations of four distinct bacteria (*Pseudomonas yamanorum*, *Cutibacterium acnes*, *Ralstonias insidiosa*, and *Pseudomonas antarctica*) in samples of gastric secretions, saliva, serum, and urine all showed a statistically significant increase in patients with gastric dysplasia and gastric cancer compared with controls (81). In another, an increase in organisms more typically found in the oral microbiome (*S. odonotylitica, S. cristatus*, and *P. stomatis*) was also observed in saliva, gastric secretions, and gastric tissue samples from patients with gastric adenocarcinoma (82).

### Pancreatic cancer

Multiple studies have found that not only does the pancreas harbor a diverse microbiome, but that dysregulation of this microbiome has significant implications for the development and progression of adenocarcinoma, with more diverse microbiomes correlated with improved long-term survival (72). Conversely, microbiomes that showed increased levels of *Proteobacteria, Mycoplasma, Fusobacterium nucleatum, Actinobacteria*, and *Verrucomicrobia* were associated with pancreatic cancer development (70–72). Additionally, in mouse trials, *Proteobacteria* were most strongly correlated with advanced disease, while *Bifidobacterium pseudolongum* has been found to accelerate oncogene expression in a TLR-dependent manner (135). Most intriguingly, in one study comparing the intratumoral microbiomes of patients with pancreatic cancer who had long-term survival (LTS) versus short-term survival (STS), STS tumors had significantly lower microbial diversity than LTS tumors. When these intratumoral microbiomes were compared, LTS tumors showed significantly increased presence of *Saccharopolyspora*, *Pseudoxanthomonas*, *Streptomyces*, and *Bacillus clausii* (88). This difference in microbiota was further validated in a mouse trial in which samples of STS and LTS tumor microbiomes, along with tissue microbial samples from healthy controls (HC), were introduced into PDAC model mice via fecal microbiota transplant (FMT), and found that mice with introduced LTS microbiome had significantly improved outcomes compared to those with introduced STS or HC microbiomes and that the tumors in the LTS transplanted mice had improved infiltration of immune cells, particularly CD8 T-cells (88). This result suggests a connection between the intratumoral microbiome and response to chemo or immunotherapy.

### Esophageal cancer

Esophageal cancer can be divided into two subsets, esophageal adenocarcinoma (EAC) and esophageal squamous cell carcinoma (ESCC). Given the geographic distribution, it is unsurprising that there is overlap in microbial changes across both subsets, as populations exposed to similar environmental factors, diets, and microbial ecologies may show shared shifts in the esophageal microbiome (73). Both subsets have been found to harbor elevated levels of bacteria associated with oral dysbiosis, including *Prevotella oris, Prevotella melaninogenica*, and *Veillonella parvula*. *Fusobacterium nucleatum* has also been detected in esophageal tumor biopsies, and its increased presence has been correlated with poorer outcomes. However, distinct differences have emerged between these subsets as well (70, 72). EAC has been found to have increased concentrations of *Lactobacillus* and *Enterobacteria* species, with decreased concentrations of *Siphonobacter, Algae, Nitrobacter, and Planckia* species (109).

Additionally, the presence of *H. Pylori* in esophageal tissue has been correlated with a reduced incidence of EAC. ESCC, on the other hand, has been found to contain increased *Porphyromonas gingivalis* and *Streptococcus anginosus*, and significant decreases in *Faecalibacterium*, *Bacteroides*, *Curvibacter*, and *Blautia* (109, 110). Given that these cancers share a relatively close geographic distribution but have significantly different risk factors and pathogenesis, additional research will be needed to study them separately and to continue identifying differences in their intratumoral microbiomes.

### Hepatocellular carcinoma

Hepatocellular Carcinoma (HCC) has long been linked with chronic inflammation, with a variety of common causes being chronic hepatitis infections, alcohol, or metabolic disorders (136, 137). However, recent studies have shown that the intratumoral and surrounding tissue microbiomes may also play a role in the development of this malignancy. At the intratumoral level, HCC was found to have higher concentrations of *Actinobacteria, Proteobacteria*, and *Firmicutes* than surrounding non-cancerous liver tissue. In comparison, patients with tumors containing higher concentrations of *Akkermansia* and *MMM* had improved overall survival (92). One study found that patients with HCC who had stool samples showing increased abundance of *Proteobacteria* species were more likely to be non-responders to anti-PD-1 therapies, and an increased concentration of *Clostridium scindens* appeared to decrease the accumulation of natural killer T-cells in the liver, thereby increasing the risk of metastasis (11, 71, 138) Additional studies of fecal microbiota in patients with HCC have revealed other changes, including increased *Ruminococcaceae*, although the risk factor for this change remains unknown (139).

### Intrahepatic cholangiocarcinoma

Studies on the intratumor microbiome of intrahepatic cholangiocarcinoma (ICC) have shown significant differences between groups that are sensitive or resistant to chemotherapy drugs gemcitabine and cisplatin, which are part of the standard of care for treatment of non-resectable ICC (86). Both groups showed increased levels of *Gammaproteobacteria, Actinobacteria*, and *Alphaproteobacteria*, but the concentration of *Gammaproteobacteria* was even higher in the treatment-resistant group (139). Other research has shown that increased levels of *Paraburkholderia fungorum* in the tissue surrounding the tumor are associated with decreased tumor growth and are thought to cause this inhibition due to activity in metabolizing alanine, aspartate, and glutamate in the local environment, preventing these amino acids from being taken up by tumor tissue (87). There has also been evidence showing that bacteria in the intratumoral environment translocate from the bile microbiota, indicating that the bile microbiome may have significant impact on the course of a given cholangiocarcinoma, and demonstrated that the combination of *Sphingomonas, Staphylococcus, Massilia, Paenibacillus, Porphyrobacter, Lawsonella*, and *Aerococcus* had predictive value in cholangiocarcinoma diagnosis, staging, and prognosis, with bile *Staphylococcus* alone as a possible predictor of the presence of cholangiocarcinoma (140). Of note, while investigation into the intratumoral microbiome of ICC has begun, there is little to no evidence for the effect of the microbiome on extrahepatic cholangiocarcinoma (ECC) or other cholangiocarcinoma subsets.

### Lung cancer

Lung cancer is a largely heterogeneous malignancy but can primarily be divided into Small Cell Lung Cancer (SCLC) and Non-Small Cell Lung Cancer (NSCLC). NSCLC can further be subdivided into adenocarcinoma, squamous cell carcinoma, and large cell carcinoma. In one study, the presence of *Escherichia* in the intratumoral environment was associated with improved survival in patients with NSCLC who received immune checkpoint blockade (ICB) therapy, but not in those treated with chemo-immunotherapy (96). Although the exact mechanism underlying this result is unknown, it is hypothesized that *Escherichia coli* is linked to the upregulation of multiple genes involved in immune cell recruitment into the tumor, including GZMB, CCL20, CXCR2P1, CXCL13, and IL12RBR (96, 141–146). In another study that compared the microbiota of two distinct lung adenocarcinoma subtypes with distinct radiological findings, the less aggressive subset was found to have greater intratumoral microbiome diversity, including elevated *Chloroflexi, Gemmatimonadetes, Cloacibacterium, Subdoligranulum*, and *Mycobacterium* (97). When lung adenocarcinoma tissue was compared directly with normal lung tissue, the malignant tissue was found to have higher levels of *Actinobacteria* and *Proteobacteria.* At the same time, the concentrations of *Firmicutes* and *Bacteroidetes* decreased, and a further association of *Parvibaculum, Renibacterium*, and *Ancylobacter* with lung cancer was observed. In contrast, increased *Lactobacillus* levels were observed in normal lung tissue (97). In yet another study, comparing tissue from patients with lung cancer recurrence and those without (primarily with lung adenocarcinoma), the proportion of *Firmicutes* and *Roseburia* was increased. The proportions of *Proteobacteria* and *Actinobacteria* decreased in patients with recurrence, in both tumor and normal tissues, compared with the non-recurrence group (97).

### Breast cancer

Examination of the intratumoral microenvironment in breast cancer has revealed several unexpected findings. In one study that compared breast tumor tissue against tumor tissues from lung, ovary, pancreas, melanoma, bone, and brain, it was found that breast cancer had the highest microbial diversity, with an average of 16 bacterial species in breast cancer compared to 9 or fewer in the other cancer sets (17) Another study comparing the microbial environments of malignant breast tumors, benign breast tumors, and standard breast tissue found that breast cancer tissue harbored a higher abundance of *Enterobacteriaceae* and *Staphylococcus*, bacterial groups that have been associated with DNA damage, including induction of double-stranded breaks, in experimental models (147). A meta-analysis showed that breast cancer also showed enrichment of *Actinomyces, Propionicimonas, Lactobacillus, Fusobacterium, Gluconacetobacter, Bartonella, Paracoccus, Hydrogenophaga, Neisseria, Citrobacter, Coxiella*, and *Escherichia Coli*, when compared to breast tissue or breast milk (99). Additionally, it has been found that in triple-negative breast cancer, tumors with increased intratumoral microbiome diversity had increased TME activity that correlated with improved response to chemo/immunotherapy (24). Lastly, in mouse models, it was demonstrated that intracellular microbiota within the tumor microbiome played a key role in promoting tumor metastasis, particularly in tumors enriched with *Staphylococcus* and *Lactobacillus* (148).

### Cutaneous melanoma

In one study comparing the intratumoral and skin microbiomes in pigs, it was found that melanoma samples contained *Firmicutes, Fusobacterium*, and *Trueperella*, and showed a higher abundance of *Staphylococcus* and *Streptococcus* than standard skin samples. Interestingly, the quantity of *Fusobacterium nucleatum* increased with the age of the melanoma (127). In another study, the presence of certain bacterial species, including *Cutibacterium granulosum, Cutibacterium acnes*, and *Corynebacterium kefirresidentii*, correlated with lower recurrence rates and improved survival. In contrast, the presence of *Leuconostoc inhae, Streptococcus salivarius, Collinsella*, and *Porphyromonas* was associated with increased recurrence and decreased survival (128). In another study, increased levels of *Lachnoclostridium* correlated with increased CD8^+^ T-cell penetrance, and patients with tumors with high CD8+ T-cell penetrance had improved overall survival (21) (**Figure 1**).

### Head and neck squamous cell carcinoma

In one bioinformatic analysis, HNSCC was found to contain high concentrations of *Fusobacterium*, *Prevotella*, and *Streptococcus* (113). These were also found to correlate with environmental risk factors for HNSCC. In patients who did not have a history of alcohol use or smoking, elevated levels of *Fusobacterium* were associated with a good prognosis. In contrast, high levels of *Streptococcus* were correlated with poor outcomes in patients who had no history of alcohol use (113). In another study that compared the geographical distribution of the tumor microbiome across different head and neck cancer sites and patients in different countries, it was found that although there was variation, *Fusobacterium*, *Prevotella*, and *Alloprevotella* were present in all groups (149). In another study, it was found that in oral squamous cell carcinoma (OSCC), tumors colonized by *Streptococcus mutans* displayed increased CD8+ T-cell exhaustion within the TME, thereby promoting further tumorigenesis of OSCC (114). In another study, HNSCC was divided into three immune subtypes: ICI-1, with an intermediate immune score and elevated fibroblast content; ICI-2, with the lowest immune score; and ICI-3, with the highest immune score (150). Tumors in the ICI-2 category had the highest microbial diversity and abundance among the three groups. They were associated with the lowest immune infiltration and the poorest prognosis (150). Specifically, ICI-2 had the highest proportion of *Fusobacterium nucleatum*, and was specifically contained *Capnocytophaga canimorsus*, *Tissierellia bacterium*, *Simonsiella muelleri, Porphyromonadaceae bacterium, and Streptococcus* sp. HMSC056D07, none of which were found in the other ICI subtypes, and of these five specific bacterial species, four were positively associated with IL-1 levels (*Tissierellia* was the exception here) (150).

### Cervical cancer

When examining the mechanism of tumorigenesis in cervical cancer, it is known that certain high-risk strains of HPV play a direct and significant role (151, 152). However, evidence has suggested that patients with increased dysbiosis of the cervicovaginal tissue are at increased risk of oncogenesis following infection with HPV, compared to those who are not in dysbiosis (153). In one study comparing cervical cancer tissue, paracancerous tissue, and normal cervical tissue, the cancerous cervical tissue showed increased levels of *Pseudomonas, Prevotella, Alistipes, Bacillus, Staphylococcus, Mycobacterium*, and *Neisseria* compared to both paracancerous and normal cervical tissues. Most specifically, patients with higher enrichment of *Pseudomonas* in the cervical tissue showed a correlation with poorer overall survival rate (107). In another study, tumors with higher *Bifidobacteriaceae* levels showed increased recruitment and activation of CD8+ cells, which correlated with improved outcomes in patients who received radiotherapy (25).

### Ovarian cancer

In one study, ovarian cancer was found to have significantly lower microbiota diversity and abundance than other tumors, with an increased ratio of *Proteobacteria* to *Firmicutes* compared with normal ovarian tissue (108). In yet another study, ovarian tumors were found to be subdivided into two different TME profiles, immune-deficient (cluster 1) and immune-enriched (cluster 2). These two clusters also showed significant differences in their microbiomes, with cluster 1 containing a significantly higher proportion of *Pseudomonas*. Other bacteria found in cluster 1 associated with lower overall survival (OS) and progression-free survival (PFS) included *Klebsiella michiganensis, Buchnera aphidicola, Paraburkholderia edwinii, Comamonas aquatica, Veillonela nakazawae, Corynebacterium jeikeium*, and *Bifidobacterium subtile*. In cluster 2, *Gemmatirosa kalamazoonesis* was significantly associated with increased OS and PFS (36). In another study, tumors with high abundance of *Simiduia*, *Halolamina*, *Brachymonas*, and *Terasakiella* were associated with improved OS. In contrast, a high abundance of *Magnetospirillum, Luteimonas, Mitsuokella, Erwinia*, and *Saliniesphaera* was correlated with decreased OS (125). Together, these studies suggest that more research is needed to understand the interactions of this microbiome and the complexities of this tumor environment.

### Uterine and endometrial cancer

In one study that compared the tumor tissue with paracancerous tissue, it was found that endometrial cancer tissue had proportionally lower *Firmicutes* and increased abundance of *Bacteroidota*, *Fusobacteria*, and *Campilobacterota* (100). Alternatively, in another study that utilized a scoring system called Resident Microbiome of the Endometrium (RME), they found nine bacterial species that affected OS, with seven (*Zooshikella, Caldimonas, Candidatus Accumulibacter, Nonlabens, Roseiflexus, Steptosporangium*, and *Zavarzinella*) correlated with a favorable OS, and two (*Derxia* and *Shigella)* were correlated with poorer survival (74). By categorizing endometrial and uterine tumors by their Relative Microbial Enrichment (RME) score, with a lower score correlating with improved survival, they also found that tumors with low RME scores had significantly more infiltration by immune cells than tumors with high RME scores (74). In yet another review, it was found that increased abundance of *Nitriliruptor* and *Blautia* also correlated with poor prognosis in this malignancy (101).

### Prostate cancer

In one study, the microbiome of localized prostate cancer and advanced prostate cancer was compared, with significant differences seen between the two. Advanced prostate cancer had significantly less microbial diversity, and in particular, localized prostate cancer was enriched with *Enhydrobacter*, while advanced prostate cancer was significantly enriched in *Lautropia*. Interestingly, when used for *in vitro* studies, the introduction of *Lautropia* in cell culture resulted in an increase in cell proliferation (105). In another study, the bacteria associated with Gleason scores, TMN staging, and PSA levels were examined, with surprising differences between these groups (106). *Pedicoccus pentosaceus, Listeria monocytogenes*, and *Lactobacillus crispatus* were found to be negatively associated with Gleason score and positively associated with indolent cancer progression; *Methybacterium radiotolerans, Stenotrophomonas maltophilia*, and *Pseudomonas aeruginosa* were negatively correlated with TMN staging; *Campylobacter concisus, Thermos thermophilus*, and *Streptococcus pneumoniae* were positively correlated with elevated PSA levels, while *Xanthomonas albilineans, Herminiimonas arsenicoxydans*, and *Pseudoarthrobacter chlorophenolicus* were all strongly negatively correlated with PSA levels (106). This study also investigated microbial regulation of the androgen receptor gene and found that tumors with low androgen receptor had increased levels of *Bradyrhizobium elkanii, Ochrobactrum anthropi*, and *Bradyrhizobium japonicum*, while tumors with high androgen receptor levels contained an abundance of *E. Coli* (106).

### Urothelial (bladder) cancer

Of all cancers, bladder cancer is one of the most closely affiliated with the idea of microbial interactions affecting tumor progression, as intravesicular *Bacillus Calmette-Guérin (BCG)*, a live attenuated strain of *Mycobacterium bovis*, has been used as part of the standard of care treatment regimen and is thought to function by upregulating infiltration of the immune system into the cancerous tissue (154). However, research into the intratumoral microbiome is still in its early days. In one limited study, two out of ten patients were found to have tumors with a biofilm layer of *E. coli*, and neither patient had a history of infection with *E. coli* (126). In a larger study, patients were divided into groups based on tumoral expression of epithelial-mesenchymal transition (EMT)-related genes (high, medium, and low) and compared their intratumoral microbiome profiles. EMT high tumors were associated with *Terrabacter, Acinetobacter*, and *Lachnoclostridium*; EMT medium tumors were associated with *Lactobacillus, Bacillus*, and *Mycobacterium*; EMT low tumors were associated with *Acidibacillus* and *Cyanothece*. More specifically, it was found that *Lachnoclostridium* and *Sutterella* promoted EMT, increasing the likelihood of tumor expansion through the epithelium into the bladder mesenchyme (126). *Lachnoclostridium* was further studied, and in another study, tumors enriched with this bacterial genus showed increased chemokine release and macrophage and CD8^+^ T-cell recruitment, with significant implications for this microbe’s impact on the response to immunotherapy (24).

### Renal cell carcinoma

Although there are multiple subtypes of kidney cancer, the most common subtype is Renal Cell Carcinoma (RCC). In one study comparing the microbiomes of RCC tumors with those of non-cancerous renal tissue, decreased species diversity was observed in RCC tissue. *Cyanobacteria* were found to be more concentrated in the normal tissue, while *Deinococcus* was found to be more concentrated in cancerous tissue (121). In another study, six intratumoral bacterial genera were found to be associated with a favorable prognosis, including *Plasmodium, Babesia, Toxoplasma, Cytobacillus, Alicyclobacillus*, and *Verrucomicrobium. In comparison*, an additional four intratumoral bacterial genera were found to be correlated with negative prognosis, including *Colletotrichum, Leuconostoc, Gluconobacter*, and *Parabacteriodes* (122). Specifically, the bacteria associated with a positive prognosis appeared to correspond with an increased anti-tumor adaptive immune response. In contrast, those associated with a negative prognosis appeared to correspond with an increased acute inflammatory response that promoted tumor growth (122).

### Adrenocortical carcinoma

This malignancy is rare and aggressive, and as such, increased knowledge of clinical response to standard of care is important. In one study, it was found that ACC tumors with high concentrations of *Proteobacteria* had better outcomes than those with low concentrations of *Proteobacteria* (124). It was also found that patients with an intratumoral microbiome abundant in *Pseudomonas* or *Serratia* and low *Firmicutes* abundance had higher therapeutic drug levels of the first-line therapy, Mitotane, which requires precise dosing due to the risk of toxic side effects (124).

### Glioblastoma multiforme

Glioblastoma multiforme is the most common primary intracranial malignancy in adults and is unfortunately very aggressive. One characteristic of this malignancy that poses a challenge for treatment is that, classically, GBM has low infiltration of T-lymphocytes, meaning it typically has a low response to immunotherapies (75). For this reason, understanding the tumor microbiome in this environment may significantly impact treatment options. In one study, the GBM microbiome was compared with the intratumoral microbiomes of other tumors (primarily breast and lung cancers) that had metastasized to the brain. The presence *of Proteobacteria, Firmicutes, Actinobacteria*, and *Fusobacterium nucleatum* was confirmed in GBM. When GBM was compared with brain metastases, it was found that metastases from other primary malignancies had significantly higher bacterial abundance and diversity than GBM, particularly a high abundance of *Lactobacillus* and *Burkholderiales*. However, it was also found that *Rhizobiales* were enriched in GBM.

Additionally, tumors that developed in the posterior brain, both GBM and metastases, had richer bacterial diversity than tumors that developed in the anterior brain (129). Other studies have begun investigating the impact of manipulating this tumor microbiome to improve the efficacy of treatment of GBM. In one study, the group demonstrated that increasing the presence of microbial peptides from specific bacteria within the GBM tissue increased T-cell migration into that tissue (38). In one *in vitro* trial, it was demonstrated that the *Staphylococcus aureus* Enterotoxin B caused a reduction of smad2/3 expression in GBM, resulting in decreased TGF-β signaling and a reduction in tumor cell proliferation (155). In another trial conducted in mice, combining *E. Coli* and anti-PD-1 therapy showed an increase in CD8^+^ T-cell upregulation in the malignant tissue (75).

The knowledge gained from our understanding of the intratumoral microbiome, summarized above, has the potential to inform the design and administration of therapeutic applications that could improve treatment outcomes for patients across many cancer diagnoses.

## Therapeutic applications

We have described how the immune landscape of a particular malignancy is anti-tumor or pro-tumor, depending in part on the effects of intratumoral microbes and the systemic microbiome, with greater diversity often skewing towards anti-tumor and lower diversity towards pro-tumor (12, 15). These observations suggest actionable targets for therapeutic design that could complement conventional chemotherapies, radiotherapies, and immunotherapies.

### Intratumoral microbiome biomarker−guided therapy selection

In recent years, it has been shown that microbial composition can predict responses across cancers. For example, intratumoral *Escherichia* associates with improved ICI outcomes in NSCLC and with increased immune cell infiltration across various tumor types (96, 100, 141–144, 146). Further, distinct microbiome signatures in HCC, RCC, ovarian cancer, and TNBC stratify prognosis and therapeutic responsiveness (102, 156, 157). Currently, many tumors are already biopsied before treatment for tumor profiling, suggesting that low−biomass sequencing of biopsies could be easily expanded to include intratumoral microbiome profiling. This information, in conjunction with other clinical findings, could inform optimal treatment strategies, as many pro-tumor microbiome profiles are associated with more aggressive cancer and resistance to conventional treatment (158). Intratumoral microbiome profiling is a key need for determining the use of other therapeutic modalities discussed in this review, and some broadly applicable options for disrupting the intratumoral microbiome already exist.

### Antibiotics

Several studies indicate that selective elimination of intratumoral microbes can restore or improve treatment sensitivity in malignancies (89, 159). In preclinical Pancreatic Ductal Adenocarcinoma (PDAC) models, targeted antibiotics restored efficacy of the chemotherapeutic gemcitabine, and in clinical series, the presence of *Klebsiella pneumoniae* tracked with outcomes and quinolone exposure (139). These data suggest that context−specific antibiotic use guided by tumor-specific microbial profiling, rather than broad-spectrum depletion of the microbiome, can improve conventional cancer therapeutic outcomes for patients. Targeted antibiotics may also be used in conjunction with probiotics to clear the pro-tumor microbiome in the area around the tumor before the addition of probiotics intended to recolonize the intratumoral microbiome.

### Probiotics and commensal enrichment

Enriching beneficial microbial taxa can enhance antitumor immunity. For example, *Akkermansia muciniphila* drives TLR2/NF−κB/NLRP3 signaling in TAMs toward M1 polarization in CRC and remodels the TME toward C2/C3−like inflamed states (22). *Bifidobacterium* species can amplify STING−dependent cytokine release, thereby promoting NK–DC crosstalk and CD8^+^ cytotoxicity in murine systems (47). These findings support the use of probiotic or symbiotic strategies tailored to tumor type and the subsequent desired immune reprogramming. Currently, no prescribable probiotic is available to physicians, much less tumor-specific probiotics that could be introduced into the intratumoral microbiome. Thus, there exists a significant gap between the primary research findings reviewed herein and actionable clinical information. While the gut microbiome can be modulated via fecal transplant, there is currently no accepted method to introduce microbiota into a tumor, which represents an avenue for future research.

### Metabolite supplementation or restriction

Microbial metabolites are potent immunomodulators that can alter therapeutic outcomes (14, 160–162). In melanoma models, *Lactobacillus reuteri*-derived indole−3−aldehyde activates AhR and skews CD8^+^ cells toward a type−1 cytotoxic phenotype, thereby improving the efficacy of immune checkpoints (115). Butyrate illustrates similar context−dependence, as it can promote lung cancer metastasis, yet in PDAC, *Clostridium butyricum*-derived butyrate increased ferroptosis susceptibility (83). These studies suggest that an approach that considers microbial metabolites, supplements beneficial metabolites, or inhibits deleterious ones could be tailored to tumor and immune contexts to promote an anti-tumor microbiome profile. However, this has several limitations, including the need to profile the tumor’s metabolites in addition to the intratumoral microbiome. Another limitation associated with this approach is determining how to supplement or restrict metabolites, with antibiotic use being the most likely clinical course for restricting them. Supplements may be given to increase specific metabolites; these may be available systemically but may also never penetrate the intratumoral microbiome. One potential solution is to engineer bacteria that express a specific set of metabolites and are directly introduced into the tumor site, which may be the most effective way to modulate the intratumoral metabolite profile.

### Engineered and cultivated bacteria

Bacteria have been engineered and utilized for a wide range of purposes, from insulin production to transgenic and transient recombinant gene expression in plant cells (163–165). Embracing this method holds great potential for engineered bacteria to act as therapeutic agents for tumor microbiome disruption and programming. Several clinical-stage companies, such as Azitra and Vedanta Biosciences, are using engineered, highly selective microbiota to treat skin and intestinal diseases (166, 167). These proofs-of-concept serve as the scientific basis for engineered and cultivated bacteria that could, in the future, include strains that secrete chemokines to recruit dendritic cells and T cells; strains engineered to modulate tumor metabolism; and engineered skin commensals that elicit systemic antitumor T−cell responses against melanoma. These engineered or cultivated bacteria would allow spatially restricted immune activation within the TME while limiting potential systemic toxicities that may occur in outright immunogenic or oncolytic bacteria.

### Immunogenic and oncolytic bacteria

Tumor−colonizing bacteria can function as danger signals that activate innate and adaptive immune responses, improving responses to immunotherapies or direct killing of tumor cells (168). Such approaches may be particularly attractive for “immune−desert” (IE−low) TMEs. These immunogenic bacteria could be specifically engineered for implantation within the intratumoral microbiome, not only disrupting the microbiota present but also directly promoting immune cell activation. Oncolytic bacteria may also fill a similar niche by directly recruiting immune cells through cytokine release; these bacteria could disrupt the intratumoral microbiome and kill tumor cells, as oncolytic viruses do (169). Unlike engineered and cultivated bacteria, which disrupt the intratumoral microbiome and would most likely be used in conjunction with current immunotherapies, oncolytic and immunogenic bacteria could serve as a differentiated therapeutic modality without the need for coadministration with more conventional modalities. This, however, makes immunogenic and oncolytic bacteria more likely to cause adverse events.

### Enzyme/virulence targeting

Disabling microbial oncogenic functions is another axis for potential intratumoral microbiome-related treatment. For example, blocking colibactin production or its epithelial adhesin requirement could mitigate genotoxicity in CRC, and inhibiting *Fusobacterium nucleatum* Fap2 interactions with TIGIT/CEACAM1 may relieve direct suppression of NK and T cells (76, 131). These are tractable protein–protein or pathway targets that lend themselves to small−molecule, peptide, or other biologic development. Ultimately, targeting these pathways may disrupt the pro-tumor skewing of the intratumoral microbiome, leading to either a neutral or an anti-tumor-skewed profile.

### Epigenetic modulation by microbes

Intratumoral taxa correlate with, and can drive, DNA methylation changes in stomach adenocarcinoma, with *Staphylococcus saccharolyticus* abundance associated with methylation of ZNF215 and increased proliferation (80). Targeting microbe−induced epigenetic reprogramming, with DNMT or HDAC inhibitors selected for the specific methylome shift, also serves as a promising approach for disrupting the pro-tumor microbiome (80).

## Discussion

The above evidence, when taken together, paints a clear picture that the intratumoral microbiome, and to some extent the systemic microbiome, has profound effects on the TME. Therefore, in future studies of the TME, microbial communities within the intratumoral microbiome should be included as a potential modifying factor that can either drive tumor resistance to therapeutic modalities or drive an anti-tumor TME with a positive response to therapies. The collective findings across tumor types further demonstrate that the intratumoral microbiome functions as a dynamic “fourth axis” of the TME, influencing immune infiltration, stromal remodeling, metabolic gradients, and therapeutic responsiveness. Microbial taxa shape the immunologic identity of tumors, influencing whether they adopt an inflamed, therapy-sensitive phenotype or an immunosuppressed, therapy-resistant state through mechanisms such as modulation of CD8^+^ and NK cell recruitment, macrophage and neutrophil polarization, extracellular matrix remodeling, and antigen presentation via HLA molecules. Together, these data emphasize that tumor-resident microbes are not simply bystanders but engaged contributors in directing cancer progression and treatment outcomes.

Building on this overarching framework, our review indicates that the intratumoral microbiome intersects the tumor, immune, and stromal compartments in ways that are both mechanistically diverse and amenable to therapeutic manipulation. Across the cancers reviewed, specific taxa and their metabolites can recalibrate TME programs aligned with established immunologic subtypes, thereby holding the potential to condition sensitivity to chemotherapy, radiotherapy, and immunotherapy (7, 17, 73). These effects arise via microbial metabolite signaling (tryptophan and short-chain fatty acids), engagement of innate pathways (STING/IFN-I), shifts in myeloid polarization (M1 versus M2 TAMs), altered DC cross-presentation, and remodeling of CAF/ECM dynamics that modulate trafficking barriers and drug penetration (39, 69, 115). Recognizing the microbiome as an integral axis of the TME, therefore, strengthens the rationale for incorporating microbial profiling and manipulation into precision oncology pipelines.

Translationally, several therapeutic avenues emerge and logically connect to the immune and stromal mechanisms summarized above. First, direct modulation can be leveraged both subtractively and additively. Targeted antibiotics may deplete resistance-promoting taxa (*Gammaproteobacteria* that inactivate gemcitabine in PDAC). In contrast, enrichment of beneficial organisms such as *Akkermansia* or *Bifidobacterium* can drive M1 macrophage polarization, enhance NK/DC crosstalk, and bolster CD8^+^ infiltration (36, 139) Metabolite-aware strategies should consider context-specific effects, such as indole derivatives from *Lactobacillus reuteri*, which can enhance cytotoxic programs through AhR in CD8^+^ T cells, or butyrate, which can either promote ferroptosis (PDAC) or facilitate metastasis (lung) (115). Engineered and cultivated bacteria may be used to deliver cytokines, chemokines, and immune potentiators in a spatially restricted manner, converting immune-desert TMEs into immune-enriched states with improved checkpoint responsiveness. These actionable treatment strategies, as well as the others discussed above, represent new avenues of embracing the intratumoral microbiome as a lever that may be influenced to increase the efficacy of established therapeutic modalities or as the basis for a new class of intratumoral microbiome-targeting therapeutics.

Despite the promise, important knowledge gaps remain, and acknowledging them guides the next steps in developing intratumoral microbiome-targeting therapies. First and foremost, causality is not fully established for many microbiome associations; for this reason, interventional trials using selective antibiotics, rational probiotic consortia, synthetic strains, or metabolite modulation should incorporate serial, contamination-aware intratumoral sampling to define mechanisms and the optimal timing relative to systemic therapy. The relationship between tumor-resident microbes, adjacent tissues, and systemic compartments remains poorly understood, and further study is needed to understand how regional ecosystems seed or sustain intratumoral communities. Secondly, there is a risk of low biomass contamination due to a lack of standardization in sampling or testing methodologies. Current methods often lack the specificity needed to provide confident profiles of microbial communities (158). However, improvements in spatial and single-cell multi-omics have outperformed standard methodologies, including 16S rRNA amplicon sequencing along with bulk metagenomics within intratumoral environments. Improvements in isolation, culture, and contamination exclusions continue to provide better aseptic conditions for intratumoral bacteria isolation (170). Moreover, reliable biomarkers are needed to predict when a given metabolite axis will exert pro− versus anti−tumor effects in a specific TME. Standardization of low-biomass sequencing and spatial analytics is therefore essential to ensure reproducibility and clinical utility.

Equally critical is situating microbiome-directed strategies within the broader determinants of tumor behavior and patient physiology. Tumor−intrinsic genetics and epigenetic states influence how microbial cues are interpreted, shaping antigen presentation, interferon responsiveness, and stromal remodeling (80). Host immune status (including immunocompromised states and immunosuppressive therapies) can amplify or blunt the impact of microbial modulation. TME biophysics (hypoxia, acidosis, nutrient competition, etc.) may limit the efficacy of intratumoral microbiome treatments unless addressed, and prior standard-of-care treatments (chemotherapy, radiation, antibiotics, etc.) can remodel local and systemic microbial ecology, affecting subsequent colonization by engineered strains or the availability of key metabolites. Safety considerations, ranging from off-target microbiome disruption to control of engineered organisms, must be built into clinical designs through tumor targeting, real-time pharmacokinetic, and spatial monitoring.

Taken together, integrating the intratumoral microbiome into cancer biology reaffirms current trends in therapeutic design from single-target interventions toward coordinated, multi-axis treatment. By aligning microbial depletion or enrichment, metabolite tuning, and programmable bacterial delivery with ICB, cytotoxic agents, and radiotherapy, it may be possible to convert immune-excluded or immune-desert tumors into immune-enriched, therapy-responsive ecosystems. Appreciating the intratumoral microbiome as a dynamic and functional component of the TME thus underscores a practical path toward more durable responses across diverse cancers, both for standard-of-care interventions and for a future of novel microbiome-modulating therapies.
